# Ghosting sexuality: A qualitative study on sexual interest among young adults

**DOI:** 10.1192/j.eurpsy.2026.12051

**Published:** 2026-07-31

**Authors:** S. von Humboldt, G. Low, I. Leal

**Affiliations:** 1 William James Center for Research, ISPA – Instituto Universitário, Lisbon, Portugal; 2 Faculty of Nursing, International Health Research, MacEwan University, Edmonton, AB, Canada

## Abstract

**Introduction:**

The decline in sexual interest among young adults is a growing phenomenon with profound implications for their relationships, mental health, and overall well-being.

**Objectives:**

This qualitative study aims to: (a) investigate how young adults emotionally experience changes in sexual interest and (b) examine the link between sexual interest and mental health.

**Methods:**

A total of 651 individuals aged between 18 and 30 years (*M* = 25.2; *SD* = 2.47), representing Portuguese, Brazilian, and English nationalities, participated in this study. Data were collected through in-depth semi-structured interviews and examined using content analysis.

**Results:**

For the first objective, eight themes emerged: Reduced face-to-face interaction (87.7%); Challenges in navigating online dating (82.3%); Negative past sexual encounters (77.1%); Sexual initiation at an early age (73.0%); Negative body image perceptions (68.2%); Frequent engagement with social media (66.8%); Consumption of pornographic content (57.3%); and Digital exhaustion from remote work (55.8%). For the second objective, four themes highlighted: Increased stress levels (92.5%); Presence of depressive symptoms (88.6%); Experience of social anxiety (79.7%); and Emergence of dependency patterns (71.7%). Portuguese participants emphasized difficulties related to reduced face-to-face interactions and stress, while Brazilian participants emphasized online-related challenges and social anxiety. In contrast, English participants highlighted the impact of past experiences and social anxiety, reflecting distinct cultural influences on both sexual desire and mental health.

**Conclusions:**

This study uncovers how intertwined social, digital, and emotional elements contribute to reduced sexual interest in young adults. Addressing emerging mental health concerns and evolving social expectations is crucial for promoting healthier relationships and overall well-being in this demographic.

Keywords: Mental health; qualitative study; sexual interest; sexual well-being; young adults.

**Disclosure of Interest:**

None Declared

